# Supplementary material for: Content-rich biological network constructed by mining PubMed abstracts
Source: BMC Bioinformatics. 2004 Oct 8;5:147. doi: 10.1186/1471-2105-5-147 (PMC528731; doi:10.1186/1471-2105-5-147)
Supplement: Additional File 2 — The original results of the above study (non-essential files are deleted to keep the file size under the limit set by BMC bioinformatics). [file 1471-2105-5-147-S2.bz2 › chilibotAdditionalFile2/dip10/308ID10508860E1337/html/RPL10_PSEN1.html]

 


 **RPL10** and **PSEN1** 
  
Found 5 abstracts in PubMed, retrieved 5.  
 

 What does Google say? 
 PDF only 
| .edu only 

---

- Int J Syst Evol Microbiol, 2003   **Pseudomonas koreensis sp.
  nov **[RPL10]**.
  , Pseudomonas umsongensis sp.
  nov **[RPL10]**.
  and Pseudomonas jinjuensis sp.
  nov **[RPL10]**.
  , novel species from farm soils in Korea.** .
  Among Pseudomonas strains isolated from Korean agricultural soils, four strains Ps 9 14 group Ps 1 **[PSEN1]** 2, Ps 1 **[PSEN1]** 10, Ps 5 5 and Ps 9 14T from the Suwon, Goesan and Samchok regions, three strains Ps 3 10 group Ps 2 22, Ps 3 1 and Ps 3 10T from Umsong Region and four strains Pss 26 group Pss 14, Pss 25, Pss 26T and Pss 27 from Jinju Region were identified as three independent groups on the basis of 16S rDNA sequence analysis.
  While, on the basis of 16S rDNA sequence analysis, Ps 9 14T and Ps 3 10T form a phyletic line with Pseudomonas jessenii CIP 105274T, Pseudomonas pavonaceae IAM 1155 and Pseudomonas graminis DSM 11363T, Pss 26T is grouped with Pseudomonas citronellolis ATCC 13674T and Pseudomonas nitroreducens IAM 1439T.
  According to DNA DNA hybridization studies, strain Ps 9 14T shows high DNA relatedness to strain Ps 3 10T 52% and Pseudomonas migulae CIP 105470T 49% and strain Ps 3 10T reveals high relatedness to strain Ps 9 14T 48% and P. jessenii CIP 105274T 45% .
  Strain Pss 26T shows high relatedness to P. citronellolis LMG 18378T 54% , P. nitroreducens ATCC 33634T 48% and Pseudomonas aeruginosa LMG 1242T 48% .
  On the basis of phenotypic and genotypic analyses, three novel species of the genus Pseudomonas are proposed Pseudomonas koreensis sp.
  nov **[RPL10]**.
  type strain Ps 9 14T =LMG 21318T =KACC 10848T for the Ps 9 14 group, Pseudomonas umsongensis sp.
  nov **[RPL10]**.
  type strain Ps 3 10T =LMG 21317T =KACC 10847T for the Ps 3 10 group and Pseudomonas jinjuensis sp.
  nov **[RPL10]**.
  type strain Pss 26T =LMG 21316T =KACC 10760T for the Pss 26 group.

  - Genetics, 1999   **Genetic characterization of cytological region 77A D harboring the presenilin **[PSEN1]** gene of Drosophila melanogaster.** .
    We performed a systematic lethal mutagenesis of the genomic region uncovered by Df 3L rdgC co2 cytological interval 77A D to isolate mutations in the single known Presenilin **[PSEN1]** Psn gene of Drosophila melanogaster.
    Because this segment of chromosome III has not been systematically characterized before, inter se complementation testing of newly recovered mutants was carried out.
    A total of 79 lethal mutations were isolated, representing at least 17 lethal complementation groups, including one corresponding to the Psn gene.
    Fine structure mapping of the genomic region surrounding the Psn transcription unit by transgenic rescue experiments allowed us to localize two of the essential loci together with Psn within an approximately 12 kb genomic DNA region.
    One of these loci, located 3 to Psn, encodes a Drosophila protein related to the yeast 60S ribosomal protein L10 **[RPL10]** precursor.
    We also determined which of the newly recovered lethal mutant groups correspond to previously isolated lethal P element insertions, lethal inversion breakpoints, and lethal polo gene mutants.
    Point mutations were identified in all five recovered Psn alleles, one of which results in a single amino acid substitution G E at a conserved residue in the C terminal cytoplasmic tail of the protein, suggesting an important functional role for this C terminal domain of Presenilin **[PSEN1]**.
    In addition, some viable mutations were recovered in the screen, including new alleles of the clipped and inturned loci.

    - Int J Syst Bacteriol, 1995   **Cycloclasticus pugetii gen.
      nov **[RPL10]**.
      , sp.
      nov **[RPL10]**.
      , an aromatic hydrocarbon degrading bacterium from marine sediments.** .
      Three heterotrophic bacterial strains were isolated from different locations in Puget Sound, Washington, by using biphenyl as the principal carbon source.
      These strains grow by using a limited number of organic compounds, including the aromatic hydrocarbons naphthalene, phenanthrene, anthracene, and toluene, as sole carbon sources.
      These aerobic, gram negative rods are motile by means of single polar flagella.
      Their 16S rRNA sequences indicate that they are all members of the gamma subdivision of the Proteobacteria.
      Their closet known relatives are the genera Methylobacter and Methylomonas genera of methane oxidizing bacteria , uncultured sulfur oxidizing symbionts found in marine invertebrates, and clone FL5 containing 16S ribosomal DNA amplified from an environmental source.
      However, the Puget Sound bacteria do not use methane or methanol as a carbon source and do not oxidize reduced sulfur compounds.
      Furthermore, a 16S rRNA base similarity comparison revealed that these bacteria are sufficiently different from other bacteria to justify establishment of a new genus.
      On the basis of the information summarized above, we describe a new genus and species, Cycloclasticus pugetti, for these bacteria.
      strain PS 1 **[PSEN1]** is the type strain of C. pugetti.

      - Int J Syst Bacteriol, 1994   **Taxonomic descriptions of eight new non sterol requiring mollicutes assigned to the genus Mesoplasma.** .
        Twenty mollicute strains isolated primarily from insect hosts were characterized and arranged into eight new species in the genus Mesoplasma.
        Morphological examination of the organisms by electron and dark field microscopic techniques revealed that the cells of each strain were small, nonhelical, nonmotile, pleomorphic, and coccoid.
        each cell was surrounded by a single cytoplasmic membrane with no evidence of a cell wall.
        Although the new mollicutes grew well in media containing horse or fetal bovine serum, growth in serum free or cholesterol free medium occurred only when the medium contained 0.04% polyoxyethylene sorbitan Tween 80 .
        The optimum temperature for growth was usually 30 degrees C, but multiplication generally occurred over a temperature range of 10 to 32 degrees C.
        All strains catabolized glucose.
        Most strains did not hydrolyze arginine or urea, although three related strains isolated from fireflies the strain PUPA 2T T = type strain group did hydrolyze arginine.
        The genome sizes ranged from 825 to 930 kbp, and the DNA base compositions guanine plus cytosine contents ranged from 26.5 to 31.6 mol%.
        The proposed type strains of the eight new species were not serologically related to the type strains of four other Mesoplasma species, five Entomoplasma species, 11 Acholeplasma species, and 100 Mycoplasma species and subspecies.
        Strain PS 1 **[PSEN1]** = ATCC 49582 is the type strain of Mesoplasma pleciae sp.
        nov **[RPL10]**.
        , strain PUPA 2 = ATCC 49581 is the type strain of Mesoplasma photuris sp.
        nov **[RPL10]**.
        , strain YJS = ATCC 51578 corrected is the type strain of Mesoplasma syrphidae sp.
        nov **[RPL10]**.
        , strain CHPA 2 = ATCC 49578 is the type strain of Mesoplasma chauliocola sp.
        nov **[RPL10]**.
        , strain ELCA 2 = ATCC 49579 is the type strain of Mesoplasma corruscae sp.
        nov **[RPL10]**.
        , strain GRUA 1 = ATCC 49580 is the type strain of Mesoplasma grammopterae sp.
        nov **[RPL10]**.
        , strain BARC 779 = ATCC 49583 is the type strain of Mesoplasma coleopterae sp.
        nov **[RPL10]**.
        , and strain BARC 857 = ATCC 49584 is the type strain of Mesoplasma tabanidae sp.
        nov **[RPL10]**.
